# Serum metabolomics profiling by proton nuclear magnetic resonance spectrometry of the response to single oral macronutrient challenges in women with polycystic ovary syndrome (PCOS) compared with male and female controls

**DOI:** 10.1186/s13293-023-00547-2

**Published:** 2023-09-22

**Authors:** Héctor F. Escobar-Morreale, María Ángeles Martínez-García, María Insenser, Nicolau Cañellas, Xavier Correig, Manuel Luque-Ramírez

**Affiliations:** 1 Diabetes Obesity and Human Reproduction Research Group, Department of Endocrinology and Nutrition, Hospital Universitario Ramón y Cajal, Centro de Investigación Biomédica en Red Diabetes y Enfermedades Metabólicas Asociadas (CIBERDEM), Instituto Ramón y Cajal de Investigación Sanitaria (IRYCIS), Universidad de Alcalá, Carretera de Colmenar km 9.1, 28034 Madrid, Spain; 2 Department of Electronic Engineering, Centro de Investigación Biomédica en Red Diabetes y Enfermedades Metabólicas Asociadas (CIBERDEM), Institut d’Investigació Sanitària Pere Virgili, Universitat Rovira i Virgili, Tarragona, Spain

**Keywords:** Androgens, Estrogens, Metabolism, Metabolic flexibility, Postprandial dysmetabolism, Obesity, Sex

## Abstract

**Background:**

The polycystic ovary syndrome (PCOS) is associated with insulin resistance, obesity and cardiometabolic comorbidities. We here challenged the hypothesis, using state-of-the-art proton nuclear magnetic resonance spectrometry (^1^H-NMRS) metabolomics profiling, that androgen excess in women induces a certain masculinization of postprandial metabolism that is modulated by obesity.

**Materials and methods:**

Participants were 53 Caucasian young adults, including 17 women with classic PCOS consisting of hyperandrogenism and ovulatory dysfunction, 17 non-hyperandrogenic women presenting with regular menses, and 19 healthy men, selected to be similar in terms of age and body mass index (BMI). Half of the subjects had obesity. Patients were submitted to isocaloric separate glucose, lipid and protein oral challenges in alternate days and fasting and postprandial serum samples were submitted to ^1^H-NMRS metabolomics profiling for quantification of 36 low-molecular-weight polar metabolites.

**Results:**

The largest postprandial changes were observed after glucose and protein intake, with lipid ingestion inducing smaller differences. Changes after glucose intake consisted of a marked increase in carbohydrates and byproducts of glycolysis, and an overall decrease in byproducts of proteolysis, lipolysis and ketogenesis. After the protein load, most amino acids and derivatives increased markedly, in parallel to an increase in pyruvate and a decrease in 3-hydroxybutyric acid and glycerol. Obesity increased β- and d-glucose and pyruvate levels, with this effect being observed mostly after glucose ingestion in women with PCOS. Regardless of the type of macronutrient, men presented increased lysine and decreased 3-hydroxybutyric acid. In addition, non-obese men showed increased postprandial β-glucose and decreased pyroglutamic acid, compared with non-obese control women. We observed a common pattern of postprandial changes in branched-chain and aromatic amino acids, where men showed greater amino acids increases after protein intake than control women and patients with PCOS but only within the non-obese participants. Conversely, this increase was blunted in obese men but not in obese women, who even presented a larger increase in some amino acids compared with their non-obese counterparts. Interestingly, regardless of the type of macronutrient, only obese women with PCOS showed increased leucine, lysine, phenylalanine and tryptophan levels compared with non-obese patients.

**Conclusions:**

Serum ^1^H-NMRS metabolomics profiling indicated sexual dimorphism in the responses to oral macronutrient challenges, which were apparently driven by the central role of postprandial insulin effects with obesity, and to a lesser extent PCOS, exerting modifying roles derived from insulin resistance. Hence, obesity impaired metabolic flexibility in young adults, yet sex and sex hormones also influenced the regulation of postprandial metabolism.

**Graphical abstract:**

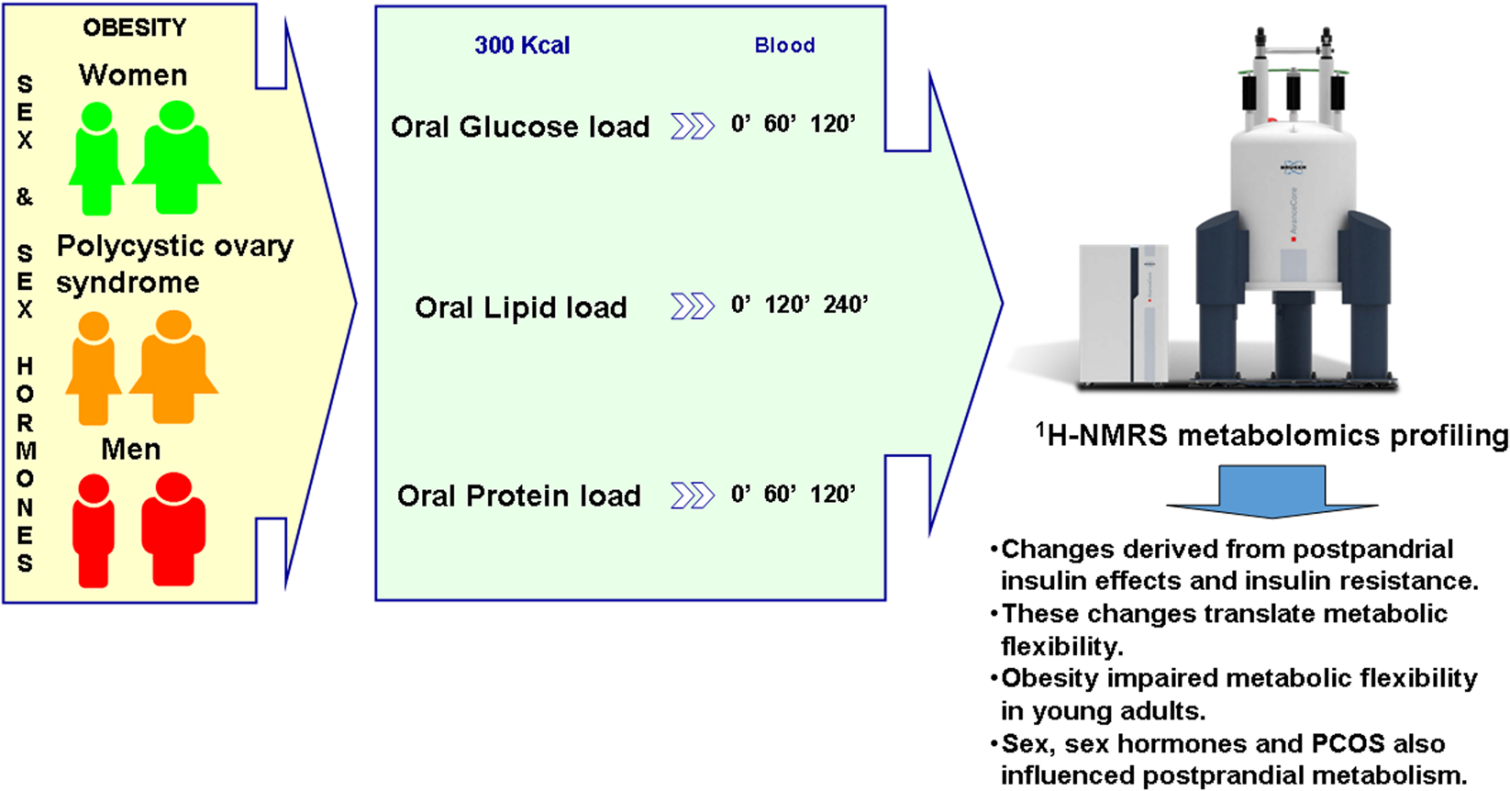

## Abstract (plain language summary)

The polycystic ovary syndrome (PCOS) is a common endocrine disorder in women. PCOS is associated with diabetes, obesity and cardiometabolic disease. Mild excess of androgens (male hormones) characterize PCOS, and facilitate that body fat accumulates in the visceral abdominal area. Visceral fat promotes insulin resistance increasing the risk for diabetes and cardiometabolic disease, and further androgen excess. We here explored intermediate metabolism after the separate administration of either carbohydrates, fats or proteins, in young adult women with or without PCOS and in men, using state-of-the-art proton nuclear magnetic resonance metabolomics profiling. Results suggest that postprandial metabolomics profiles reflect mostly insulin actions, with changes derived from insulin resistance being more important with obesity but also being influenced by male sex and PCOS in women.

## Highlights

- We studied postprandial metabolomics profiles after separate macronutrient challenges.
- Changes in these profiles derived from postprandial insulin effects and insulin resistance.
- These changes translate metabolic flexibility.
- Obesity impaired metabolic flexibility in young adults.
- Sex, sex hormones and PCOS also influenced postprandial metabolism.

## Background

The polycystic ovary syndrome (PCOS) is frequently associated with disorders of intermediate metabolism, such as visceral adiposity, obesity and type 2 diabetes [1–4]. Women with PCOS may suffer from a vicious circle from early ages, whereby hyperandrogenism may contribute to insulin resistance and hyperinsulinism by favoring visceral adiposity, and these metabolic derangements, in turn, may promote further ovarian androgen excess [5], because insulin acts as a co-gonadotropin at the ovary [6].

The serum gas chromatography–mass spectrometry metabolomics fasting phenotype of people with PCOS indicates a major role of obesity in the metabolic associations of this syndrome: while non-obese and obese women with PCOS showed evidence of central (hepatic) insulin resistance, adipose and muscle insulin resistance only develops in obese patients [7].

Sexual dimorphism in fasting and postprandial metabolism, with men usually showing worse metabolic profiles than women that are exaggerated by obesity [8–10], suggests a role for androgen excess in the metabolic derangements of PCOS [11]. Accordingly, our earlier studies showed evidence for masculinization of both adipose tissue distribution [2] and function [12–15] in women with PCOS. Moreover, recent data using proton nuclear magnetic resonance spectroscopy (^1^H-NMRS) metabolomics profiling provided evidence of sexual dimorphism and masculinization of intermediate metabolism during fasting, in women with PCOS [16].

## Materials and methods

### Aim of the study

We here aimed to investigate if ^1^H-NMRS metabolomics profiling would show evidence of masculinization of postprandial intermediate metabolism in women with PCOS, with obesity possibly exerting a modifying role.

### Subjects

Being part of a broader study addressing postprandial changes in hormonal profiles, metabolic mediators and markers of oxidative stress and inflammation in young adults (PI11/00357), a detailed description of phenotyping, protocol and methodology has been described elsewhere [10, 17–23].

The present study included 53 Caucasian young adults: 17 women with PCOS, 17 non-hyperandrogenic women presenting with regular menses, and 19 healthy men, selected to be similar in terms of age and body mass index (BMI). We classified individuals into non-obese (BMI < 30 kg/m^2^, *n* = 28) and obese (BMI ≥ 30 kg/m^2^, *n* = 25) subgroups. Total body fat mass was estimated using a body fat monitor (Omron BF 300, Omron Corp., Kyoto, Japan) and was expressed as kg and percentage of total body mass. All patients met the National Institutes of Health 1990 criteria [24] for the diagnosis of PCOS, requiring the presence of the classic phenotype consisting of clinical and/or biochemical hyperandrogenism, oligo/anovulation, and exclusion of secondary etiologies, such as hyperprolactinemia, nonclassic congenital adrenal hyperplasia and hypothyroidism. We did not include non-hyperandrogenic phenotypes of PCOS, because the study aimed to address the metabolic effects of androgen excess in women.

Control men and women had no history of hypogonadism (including male obesity-associated secondary hypogonadism), infertility or menstrual dysfunction, and no subject presented smoking habits or had received treatment with oral contraceptives, antiandrogens, sex steroids, insulin sensitizers or drugs that might interfere with clinical or biochemical variables for at least 6 months before sampling. Three men, two women with PCOS and one control woman reported having used antibiotics within 3 months prior to recruitment.

In women, hirsutism was quantified using the modified Ferriman–Gallwey score [25]. PCOS was ruled out in the control women, because all of them presented without menstrual and ovulatory dysfunction and had no evidence of clinical and biochemical androgen excess. The female and male control groups were composed of healthy volunteers recruited from the hospital's staff and of overweight or obese people seeking medical advice at our Department.

### Study design

Subjects were submitted to three oral loads of isolated macronutrients in the following order: glucose, lipids and proteins on alternate days within 1 week. Patients were instructed to follow the same written diet—containing more than 300 g of carbohydrates per day—for 3 days before sampling to avoid false positive results in the 75 g oral glucose tolerance test (OGTT), which was used not only for research purposes but also to check the patients for disorders of glucose tolerance. Hence, this test had to be the first one to be conducted and, consequently, the order of the macronutrient challenges could not be randomized. The oral loads were adjusted for a total caloric intake of 300 kcal each. We administered 200 ml of a 37.5 g/dl glucose solution in the oral glucose load (OGTT, GlycoSull Naranja 75 g, Química Clínica Aplicada, Spain), 66 ml of a 4.5 kcal/ml long-chain triglyceride enteral nutrition supplement in the oral lipid load (Supracal neutro, Nutricia S.R.L., Spain), and 75 g of an enteral nutrition supplement containing caseinates in the oral protein load (Proteína NM, Nutrición Médica S.L., Spain). The composition of the lipid supplement was 10.6%, 60.8% and 28.6% of saturated, mono- and poly-unsaturated fatty acids, respectively. All macronutrient preparations were ingested within a time-period not exceeding 5 min. After 12 h of fasting and during the macronutrient challenges, blood samples were obtained at 0, 60 and 120 min after the glucose and protein loads, and at 0, 120 and 240 min in the lipid challenge. We chose those timepoints based on our previous data and from other authors reporting that acute postprandial responses generally reached their maximum levels on such times, returning to baseline levels from there on [10, 17–20, 22]. Additional samples at 30, 90 and 120 min were taken during the OGTT for the measurement of glucose and insulin. All women were evaluated in the follicular phase of the menstrual cycle or in amenorrhea, after excluding pregnancy, and samples were immediately assayed or frozen at − 80 °C until needed.

### Hormone assays

Serum glucose was measured using the glucose oxidase method (Beckman Instruments, Indianapolis IN), and insulin was measured by direct radioimmunoassay (Diagnostic Products Corporation, Los Angeles, CA). Total *T* was measured by direct radioimmunoassay (Spectria Testosterone RIA, Orion Diagnostica Oy, Espoo, Finland) and *E*_2_, SHBG, Δ^4^A, DHEAS and hsCRP were measured using an automated immunochemiluminescence method (Immulite 2000, Siemens Healthcare Sector, Erlangen, Germany). The methods and assays used to phenotype the subjects have been reported already [26–28]. Insulin and glucose levels at fasting and during the OGTT were used to calculate homeostasis model assessment of insulin resistance (HOMA-IR) [29] and the composite insulin sensitivity index (ISI) [30], respectively. Free *T* and *E*_2_ levels were calculated from their total concentrations and SHBG levels, and the free *T* to free *E*_2_ molar ratio was calculated [31].

### Proton nuclear magnetic resonance spectrometry metabolomics profiling

Serum fasting and postprandial samples obtained during the different challenges (477 samples) were submitted to ^1^H-NMRS profiling. Thirty-six low-molecular-weight polar metabolites were identified and quantified, including branched-chain, aromatic and other amino acids and derivatives, ketone bodies, and intermediate products of glucose and lipid metabolism. First, samples were submitted to a deproteinization process aiming to remove high-molecular-weight species. Briefly, after thawing serum samples on ice, 300 µl of each sample were mixed with 1400 µl of MeOH:H_2_O (8:1) and 150 µl of IS solution in 2 ml microcentrifuge tubes, incubated at − 20 °C for 20 min and centrifuged at 15,000 rpm, at 4 °C for 10 min. After centrifugation, 1300 µl of supernatant were transferred into a 2 ml microcentrifuge tube, dried under vacuum and lyophilized overnight.

Lyophilized samples were reconstituted with 600 µl of 50 mM phosphate buffer solution (PBS) with 0.05 M TSP as internal standard, and then transferred to nuclear magnetic resonance (NMR) tube for NMR analysis. A Bruker 600 MHz Spectrometer (Bruker Biospin, Rheinstetten, Germany), was used to acquire high-resolution ^1^H-NMRS data of low molecular weight metabolites, sugars and amino acids, using 1D Carr–Purcell–Meiboom–Gill sequence, with pre-saturation to suppress the residual water peak. The acquired Carr–Purcell–Meiboom–Gill data were phased, baseline-corrected, and referenced to the chemical shift of the α-glucose anomeric proton doublet taken at 5.233 ppm, as proposed by Pearce et al. [32]. Carr–Purcell–Meiboom–Gill data were used for the profiling of the 36 metabolites, based on a new, fully automated version of the software package Dolphin [33]. Each metabolite was identified by checking for all its resonances along the spectra, and then quantified using line-shape fitting methods on one of its signals. Signal annotation was based on templates prepared in previous studies with the help of available databases [34] and bibliography [35–37]. Validation of metabolite identification was assisted by statistical total correlation spectroscopy [38]. Results are expressed as arbitrary units (a.u.) which were specific for each metabolite, precluding comparisons between different ones.

### Statistical analysis

Being this study part of a broader project aiming to address postprandial metabolism as a whole, sample size calculation was based on previous data from Gonzalez et al. [39] reporting differences between patients with PCOS and control women in the percentage change of nuclear factor kappaB expression in mononuclear cells after a standard OGTT. We used the online sample size and power calculator from the Institut Municipal d'Investigació Mèdica (Barcelona, Spain, version 7.12; https://www.imim.cat/ofertadeserveis/software-public/granmo/). Setting alpha at 0.05 and beta at 0.2 for a two-sided test, the inclusion of 8 individuals per group would allow detecting a mean difference in percentage change of 50.35%, assuming a standard deviation of 34.1%.

Data are expressed as mean ± SD (tables) or mean ± SEM (figures). Normality of continuous variables was evaluated by the Kolmogorov–Smirnov test and logarithmic transformations were applied as needed. Two-way univariate General Linear Models (GLM) were used to analyze differences in fasting variables other than metabolomics profiling, considering group of subjects (control women, women with PCOS and men), obesity, and their interaction within a single analysis.

To evaluate the responses to macronutrient ingestion we used univariate repeated-measures GLMs. We introduced the responses of individual metabolites to the different macronutrients—expressed as area under the curve (AUC)—as dependent variables. The AUC was calculated using the trapezoidal rule, corrected from fasting levels to obtain the net decrement or increment, and finally normalized by the time span of the challenge to warrant comparison between macronutrient challenges. Considering the 3 (oral glucose, lipid or protein challenges administered to all participants) × 2 (obese and non-obese persons) × 3 (control women, women with PCOS and control men) design, the type of macronutrient was introduced as within-subjects factor, and obesity (obese and non-obese subjects) and group (control women, women with PCOS and control men) were introduced as between-subjects factors. The effect of sex was estimated by comparing control women with control men; the effect of PCOS was estimated by comparing women with the syndrome with control women. Only after a statistically significant effect was found, differences between pairs of groups were identified using the least significant difference post hoc test for multiple comparisons. We used SPSS Statistics 15.0 (SPSS Inc., Chicago, IL, USA) for analyses and considered two-tailed *p* values < 0.05 as statistically significant.

## Results

### Baseline characteristics of study subjects

Table 1 shows clinical, hormonal, and routine fasting metabolic variables of participants. As expected from design, age and BMI were not different among groups. According to sex, men showed higher total and free *T* levels, waist circumference (WC), waist to hip ratio (WHR) compared with both groups of women, but had lower levels of *E*_2_, SHBG and fat mass. Women with PCOS had higher hirsutism scores and circulating androgens than non-hyperandrogenic control women, but showed no statistically significant differences in terms of WC, WHR of fat mass. In addition, we did not observe significant differences in routine metabolic variables between men and women, or control women and participants with PCOS, with the exception of lower mean high-density lipoprotein (HDL)-cholesterol values in men. Obese individuals, regardless of sex and PCOS status, showed increased total and free *E*_2_ values, free *T*, hsCRP, LDL-cholesterol, fasting glucose, insulin, and HOMA-IR, and decreased ISI, HDL-cholesterol and SHBG concentrations.

**Table 1:** Clinical, metabolic and hormonal variables in control women, patients with PCOS and men

	Control women		Women with PCOS		Control men		Group	Obesity	Interaction
	Non-obese (*n* = 9)	Obese (*n* = 8)	Non-obese (*n* = 9)	Obese (*n* = 8)	Non-obese (*n* = 10)	Obese (*n* = 9)	*P*	*P*	*P*
Age (years)	26 ± 5	27 ± 6	24 ± 8	30 ± 5	24 ± 4	25 ± 4	0.342	0.101	0.319
Body mass index (kg/m^2^)	23 ± 2	36 ± 4	24 ± 3	37 ± 5	23 ± 2	34 ± 3	0.226	**< 0.001**	0.782
Waist circumference (cm)^a,b^	76 ± 9	100 ± 17	72 ± 7	105 ± 11	81 ± 5	110 ± 13	0.050	**< 0.001**	0.377
Waist-to-hip ratio^a,b^	0.75 ± 0.08	0.83 ± 0.12	0.73 ± 0.05	0.85 ± 0.06	0.83 ± 0.04	0.90 ± 0.05	**0.002**	**< 0.001**	0.436
Fat mass (kg)^a,b^	22.5 ± 4.8	42.5 ± 10.3	20.2 ± 5.5	43.7 ± 8.6	12.9 ± 4.5	36.7 ± 12.6	**0.011**	**< 0.001**	0.767
Fat mass (%)^a,b^	35.0 ± 5.3	43.7 ± 5.7	31.6 ± 6.3	42.9 ± 3.9	16.6 ± 5.1	32.6 ± 7.0	**< 0.001**	**< 0.001**	0.165
Hirsutism score	1.4 ± 1.3	1.8 ± 1.2	9.7 ± 4.5	9.3 ± 4.5	–	–	**< 0.001**	0.738	0.876
Total *T* (nmol/l)^a,b,c^	1.6 ± 0.3	2.0 ± 0.5	2.5 ± 0.7	2.4 ± 1.0	18.5 ± 3.3	17.3 ± 3.6	**< 0.001**	0.776	0.196
Total *E*_2_ (pmol/l)^a,b^	149 ± 63	276 ± 200	182 ± 201	149 ± 49	68 ± 16	94 ± 26	**< 0.001**	**0.024**	0.422
Free *T* (pmol/l)^a,b,c^	21 ± 7	31 ± 8	36 ± 12	45 ± 24	450 ± 104	464 ± 94	**< 0.001**	**0.024**	0.265
Free *E*_2_ (pmol/l)^a^	2.7 ± 1.1	5.3 ± 3	3.6 ± 4.3	3.4 ± 1.4	1.8 ± 0.5	2.6 ± 0.7	**0.010**	**0.003**	0.520
Free *T*/free *E*_2_^a,b^^,c^	8.6 ± 0.9	7.6 ± 1.6	17.4 ± 4.0	14.0 ± 1.9	263.4 ± 22.6	186.4 ± 12.7	**< 0.001**	0.119	0.750
SHBG (nmol/l)^a,b^	56 ± 25	43 ± 14	50 ± 21	32 ± 13	27 ± 10	20 ± 6	**< 0.001**	**0.008**	0.568
Androstenedione (nmol/l)^b,c^	9.1 ± 2.9	9.5 ± 2.9	14.7 ± 4.2	13.4 ± 6.1	7.1 ± 1.7	9.3 ± 3.8	**< 0.001**	0.712	0.371
hsCRP (nmol/l)	27 ± 22	38 ± 28	20 ± 21	65 ± 74	31 ± 25	31 ± 12	0.998	**0.009**	0.207
Triglycerides (mmol/l)	0.84 ± 0.36	0.92 ± 0.34	0.90 ± 0.25	1.14 ± 0.47	0.89 ± 0.29	1.15 ± 0.40	0.399	0.054	0.711
Total cholesterol (mmol/l)	4.4 ± 0.9	4.7 ± 0.9	4.3 ± 1.0	4.4 ± 0.9	4.1 ± 0.6	4.8 ± 0.9	0.844	0.133	0.485
HDL-cholesterol (mmol/l)^a,b^	1.4 ± 0.3	1.3 ± 0.2	1.4 ± 0.2	1.2 ± 0.2	1.2 ± 0.2	1.0 ± 0.1	**0.001**	**0.002**	0.512
LDL-cholesterol (mmol/l)	2.7 ± 0.8	2.9 ± 0.6	2.5 ± 0.8	2.7 ± 0.8	2.4 ± 0.4	3.3 ± 0.8	0.507	**0.034**	0.237
Fasting insulin (pmol/l)	55 ± 20	77 ± 21	52 ± 29	94 ± 25	40 ± 11	75 ± 27	0.123	**< 0.001**	0.440
Fasting glucose (mmol/l)^b,c^	4.7 ± 0.4	5.3 ± 0.4	4.5 ± 0.5	4.8 ± 0.5	4.9 ± 0.5	5.2 ± 0.4	**0.011**	**0.001**	0.408
Insulin sensitivity index	6.6 ± 2.8	3.3 ± 1.2	8.1 ± 4.7	3.6 ± 1.4	7.3 ± 2.8	3.8 ± 1.6	0.640	**< 0.001**	0.909
HOMA-IR	1.6 ± 0.6	2.6 ± 0.7	1.5 ± 0.9	2.9 ± 0.7	1.3 ± 0.4	2.5 ± 1.0	0.422	**< 0.001**	0.744

Data are means ± SD. The effects of group and obesity were analyzed by a two-way GLM followed by the least significant difference post-hoc test

*E*_2_: estradiol; HDL: high-density lipoprotein; LDL: low-density lipoprotein; HOMA-IR: homeostasis model assessment of insulin resistance; hsCRP: high-sensitivity C-reactive protein; PCOS: polycystic ovary syndrome; SHBG: sex hormone-binding globulin; *T*: testosterone

^a^*p* < 0.05 for the difference between men and control women regardless of obesity

^b^*p* < 0.05 for the difference between men and PCOS women regardless of obesity

^c^*p* < 0.05 for the difference between PCOS and control women regardless of obesity. *P* values < 0.05 are indicated in bold

### Differences depending on the macronutrient administered

**Fig. 1:**
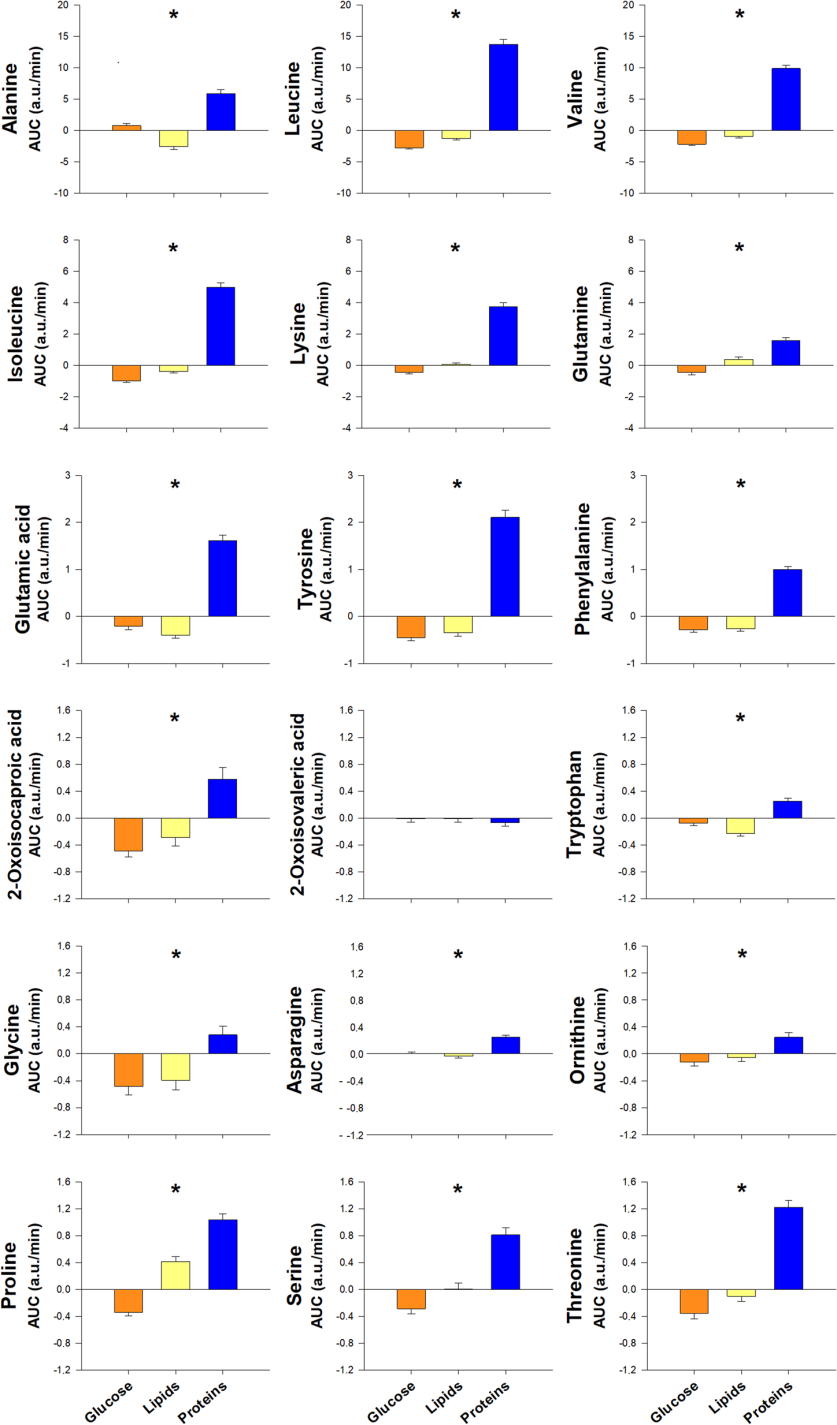

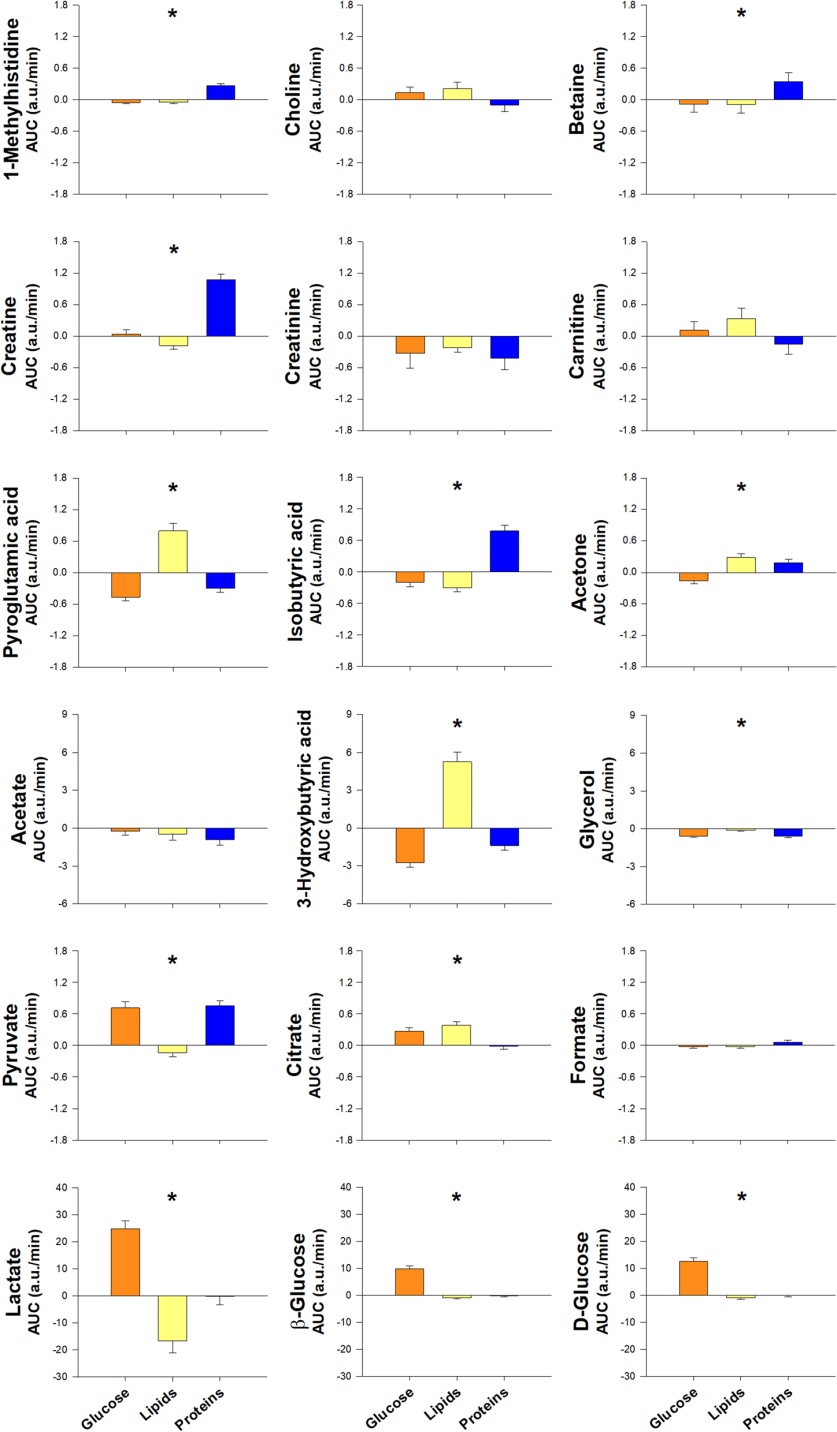
Proton nuclear magnetic resonance metabolomics profiling of amino acids, including aromatic and branched-chain amino acids and byproducts, and amino acid-derived metabolites, short-chain fatty acids, ketone bodies and carbohydrates, in response to macronutrient challenges. Data represent the AUCs (arbitrary units) as means ± SEM. Orange, yellow and dark blue colors correspond to glucose, lipid and protein loads, respectively. *p < 0.05 for the differences among macronutrient loads, considering all individuals as a whole

The largest postprandial changes in the metabolites studied here were observed after glucose and protein administration, whereas lipid ingestion induced smaller or no changes in most of them, with a few exceptions (Table 2, Fig. 1). Changes after the glucose challenge consisted of a marked increase in carbohydrates and byproducts of glycolysis such as pyruvate and lactate, and an overall decrease in byproducts of proteolysis such as amino acids, byproducts of lipolysis such as glycerol and byproducts of ketogenesis such as 3-hydroxybutyrate and acetone (Table 2, Fig. 1). Alanine was the only amino acid that increased after oral glucose ingestion (Fig. 1), probably produced by transamination of glutamate to pyruvate originated from glycolysis, forming free alanine and the tricarboxylic acid (TCA) cycle intermediate α-ketoglutarate (Table 2, Fig. 1).

**Table 2:** Summary of the effects of macronutrient loads, obesity, sex and PCOS, and their interactions, on serum postprandial metabolomics profiles

Metabolite	Glucose	Lipids	Proteins	Obesity	Sex (M vs. W)	PCOS (PCOS vs. W)	Interactions
Amino acids, including aromatic and branched-chain amino acids and byproducts							
Alanine	↑	↓↓	↑↑↑	=	=	=	
Leucine	↓	↓	↑↑↑	=	=	=	After proteins, obesity blunts the ↑ only in M After proteins, ↑ in M > W (control and PCOS) only in non-obese Only in PCOS, ↑ in obese > non-obese (loads as a whole)
Valine	↓	↓	↑↑↑	=	=	=	After proteins, obesity blunts the ↑ only in M After proteins, ↑ in M > W (control and PCOS) only in non-obese
Isoleucine	↓	↓	↑↑↑	=	=	=	After proteins, obesity blunts the ↑ only in M After proteins, ↑ in M > W (control and PCOS) only in non-obese
Lysine	↓	=	↑↑↑	=	↑	=	After proteins, obesity blunts the ↑ in M After proteins, obesity enhances the ↑ in W (control and PCOS) After proteins, ↑ in M > W (control and PCOS) only in non-obese After glucose, obesity blunts the ↓ in M After glucose, obesity enhances the ↓ in control W Only in PCOS, ↑ in obese > non-obese (loads as a whole)
Glutamine	↓	↑	↑↑↑	=	=	=	
Glutamic acid	↓	↓	↑↑↑	=	=	=	
Tyrosine	↓	↓	↑↑↑	=	=	=	After proteins, obesity blunts the ↑ in M After proteins, obesity enhances the ↑ in control W
Phenylalanine	↓	↓	↑↑↑	=	=	=	After proteins, obesity blunts the ↑ only in M After glucose, obesity blunts the ↓ in M and mainly in PCOS Only in PCOS, ↑ in obese > non-obese (loads as a whole)
2-Oxoisocaproic acid	↓	↓	↑↑	=	=	=	Obesity enhances the ↑ after proteins Obesity enhances the ↓ after glucose
2-Oxoisovaleric acid	=	=	=	=	=	=	
Tryptophan	=	↓	↑	=	=	=	Only in PCOS, ↑ in obese > non-obese (loads as a whole)
Glycine	↓	↓	↑	=	=	=	
Asparagine	=	=	↑↑↑	=	=	=	
Ornithine	↓	=	↑	=	=	=	
Proline	↓	↑	↑↑	=	=	=	
Serine	↓	=	↑↑	=	=	=	
Threonine	↓	=	↑↑↑	=	=	=	
Amino acid-derived metabolites							
1-Methylhistidine	↓=	↓=	↑↑	=	=	=	After proteins, obesity blunts the ↑ in M After proteins, obesity enhances the ↑ in W (control and PCOS) Only in PCOS, ↑ in obese > non-obese (loads as a whole)
Choline	=	=	=	=	=	=	
Betaine	↓=	↓=	↑↑↑	=	=	=	Opposite effects in PCOS vs. control W and M In PCOS: ↑ after glucose, ↓ after lipids and proteins
Creatine	=	↓	↑↑↑	=	=	=	↑ After glucose only in PCOS ↓ After lipids not in M
Creatinine	↓	↓	↓	=	=	=	
Carnitine	=	=	=	=	=	=	
Pyroglutamic acid	↓↓	↑↑↑	↓	=	↓	=	Only in non-obese, ↑ in W (control and PCOS) > M (loads as a whole)
Short-chain fatty acids, ketone bodies, glycerol and carbohydrates							
Isobutyric acid	↓	↓	↑↑	=	=	=	
Acetone	↓	↑	↑	=	=	=	Only in non-obese, ↑ in M > W (control and PCOS) (loads as a whole)
Acetate	↓=	↓=	↓	=	=	=	
3-Hydroxybutyric acid	↓↓	↑↑↑	↓	=	↓	=	
Glycerol	↓↓	=	↓↓	=	=	=	
Pyruvate	↑↑	↓	↑↑	↑	=	=	Blunted ↑ in non-obese PCOS but ↑↑ only in obese PCOS, mainly after glucose
Citrate	↑	↑↑	=	=	=	=	
Formate	=	=	=	=	=	=	
Lactate	↑↑↑	↓↓	=	=	=	=	
β-Glucose	↑↑↑	↓=	=	↑	↑	=	↑ After glucose and proteins mainly in obesity Blunted ↑ in non-obese PCOS but ↑↑ in obese PCOS ↑ In M > W (control and PCOS) only in non-obese (loads as a whole)
d-Glucose	↑↑↑	↓=	=	↑	=	=	↑ After glucose and proteins mainly in obesity Blunted ↑ in non-obese PCOS but ↑↑ in obese PCOS

↑: increase; ↓: decrease; =: no difference; M: control men; PCOS: women with polycystic ovary syndrome; W: control women. Only effects reaching statistical significance are shown

On the contrary, after the protein oral challenge most amino acids and their derivatives increased markedly, in parallel to an increase in pyruvate, isobutyrate and acetone and a decrease in 3-hydroxybutyric acid and glycerol (Table 2, Fig. 1).

Finally, the oral lipid challenge induced smaller changes or no changes in most metabolites studied here, sharing with glucose ingestion the decrease in many amino acids and derivatives (Table 2, Fig. 1). Only the amino acids glutamine and proline slightly increased after lipid ingestion. Of note, pyroglutamic acid, the ketone bodies 3-hydroxybutyrate and acetone, and the TCA cycle intermediate citrate increased in this setting, whereas glycerol was not decreased after lipid ingestion, suggesting lack of postprandial inhibition of lipolysis after lipid ingestion (Table 2, Fig. 1).

**Fig. 2:**
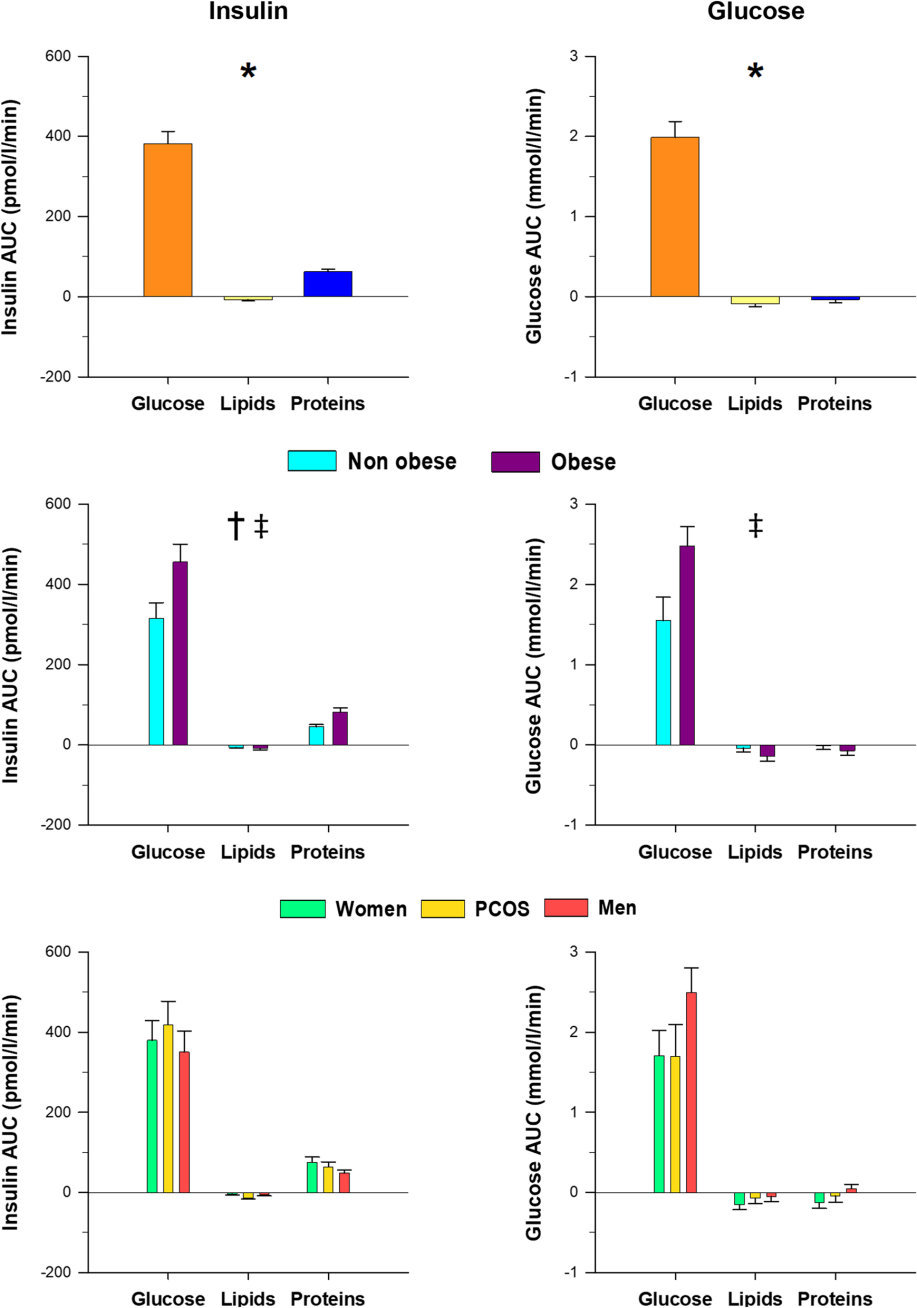
Areas under the curve of circulating insulin and glucose in response to macronutrient challenges considering all subjects as a whole, and as a function of obesity and group of subjects. Data are means ± SEM. Colors orange, yellow and dark blue correspond to glucose, lipid and protein loads; cyan and purple colors correspond to non-obese and obese individuals; and green, amber and red colors correspond to control women, women with PCOS and men, respectively. *p < 0.05 for the differences among macronutrient loads, considering all individuals as a whole. ^†^*p* < 0.05 for the differences between non-obese and obese individuals, regardless of the macronutrient ingested and group of subjects. ^‡^*p* < 0.05 for the interaction between obesity and macronutrient load, irrespective of group

Since insulin secretion was stimulated markedly after the glucose challenge and to a much lesser extent by the ingestion of proteins, but not after lipid intake (Fig. 2), the overall changes in the metabolome described here may be explained by the change from the fasting to the feed state which is characterized by the secretion of insulin and its rapid effects on intermediate metabolism, such as inhibition of proteolysis, lipolysis and ketogenesis, and stimulation of glycolysis in the liver, muscle and adipose tissue [40]. The increase in amino acids and their derivatives after protein ingestion would derive mostly from their intestinal absorption.

### Effects of obesity

**Fig. 3:**
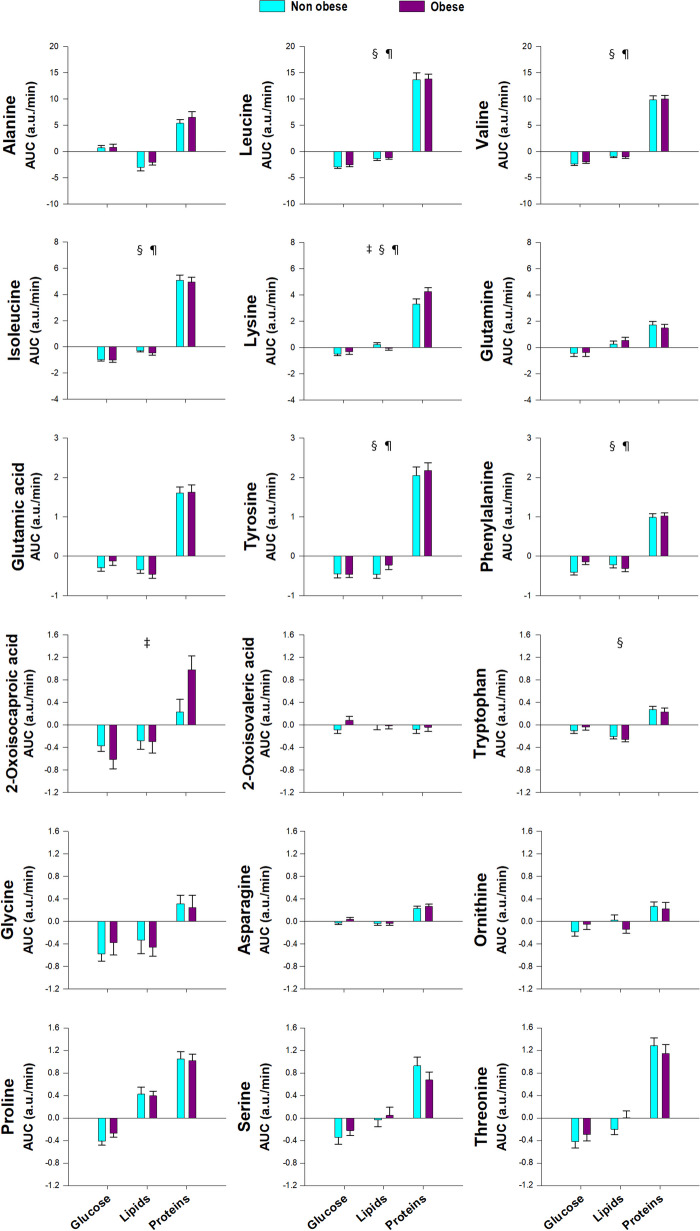

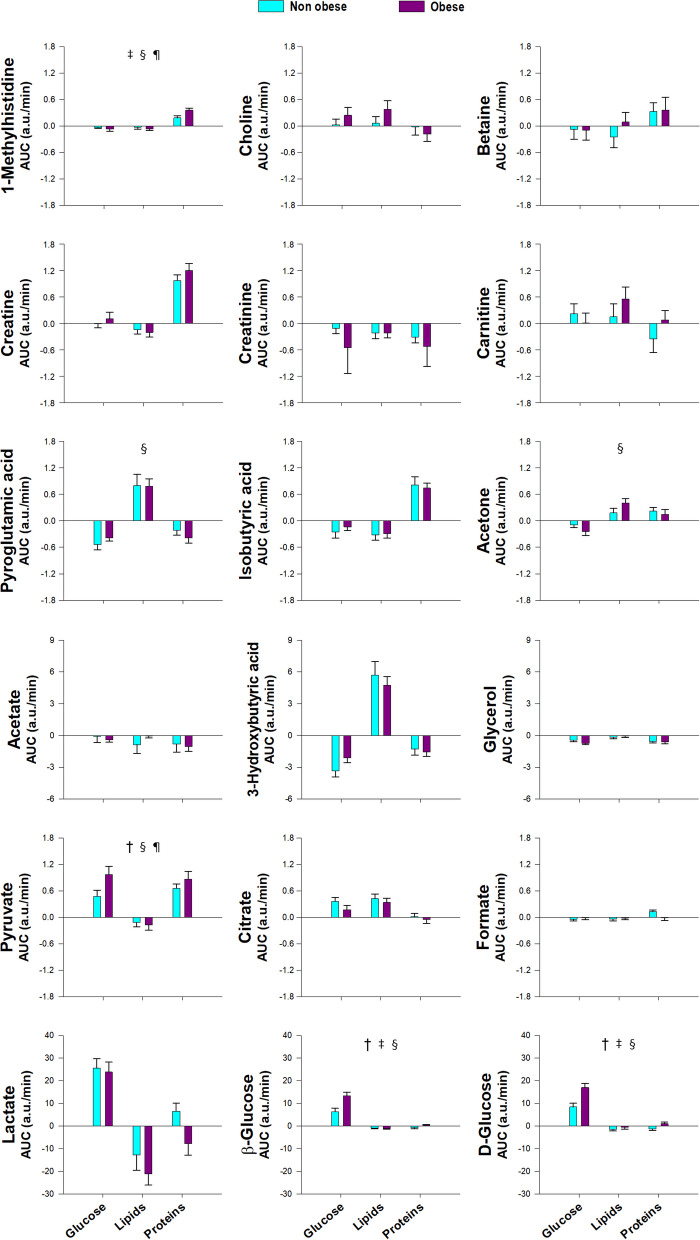
Proton nuclear magnetic resonance metabolomics profiling of amino acids, including aromatic and branched-chain amino acids and byproducts, and amino acid-derived metabolites, short-chain fatty acids, ketone bodies and carbohydrates, in response to macronutrient challenges as a function of obesity. Data represent the AUCs (arbitrary units) as means ± SEM. Colors cyan and purple correspond to non-obese and obese individuals, respectively. ^†^*p* < 0.05 for the differences between non-obese and obese individuals, regardless of the macronutrient ingested and group of subjects. ^‡^*p* < 0.05 for the interaction between obesity and macronutrient load, irrespective of group. ^§^*p* < 0.05 for the interaction between obesity and group of subjects, regardless of the macronutrient ingested. ^¶^*p* < 0.05 for the triple interaction among obesity, group and macronutrient load. ^§¶^These interactions are described in the main text and Table 2

When considering the effects of obesity in the group of subjects considered as a whole, the only effects were found on the increases in d-glucose and β-glucose and pyruvate, which were greater in obese subjects compared with their non-obese counterparts regardless of the macronutrient being ingested (Table 2 and Fig. 3). We found several interactions between obesity and the type of macronutrient ingested, consisting of the increases of d-glucose and β-glucose being much greater after glucose ingestion than after proteins and lipids, and effects of obesity in the postprandial responses of lysine, 2-oxoisocaproic acid, and 1-methylhistidine that were present only after glucose and/or protein ingestion but not when considering all macronutrients as a whole (Table 2 and Fig. 3). Again, the effects of obesity paralleled those observed on the insulin responses to the glucose and protein challenges (Fig. 2), possibly reflecting resistance to postprandial insulin effects in obese individuals.

### Differences between groups of subjects

**Fig. 4:**
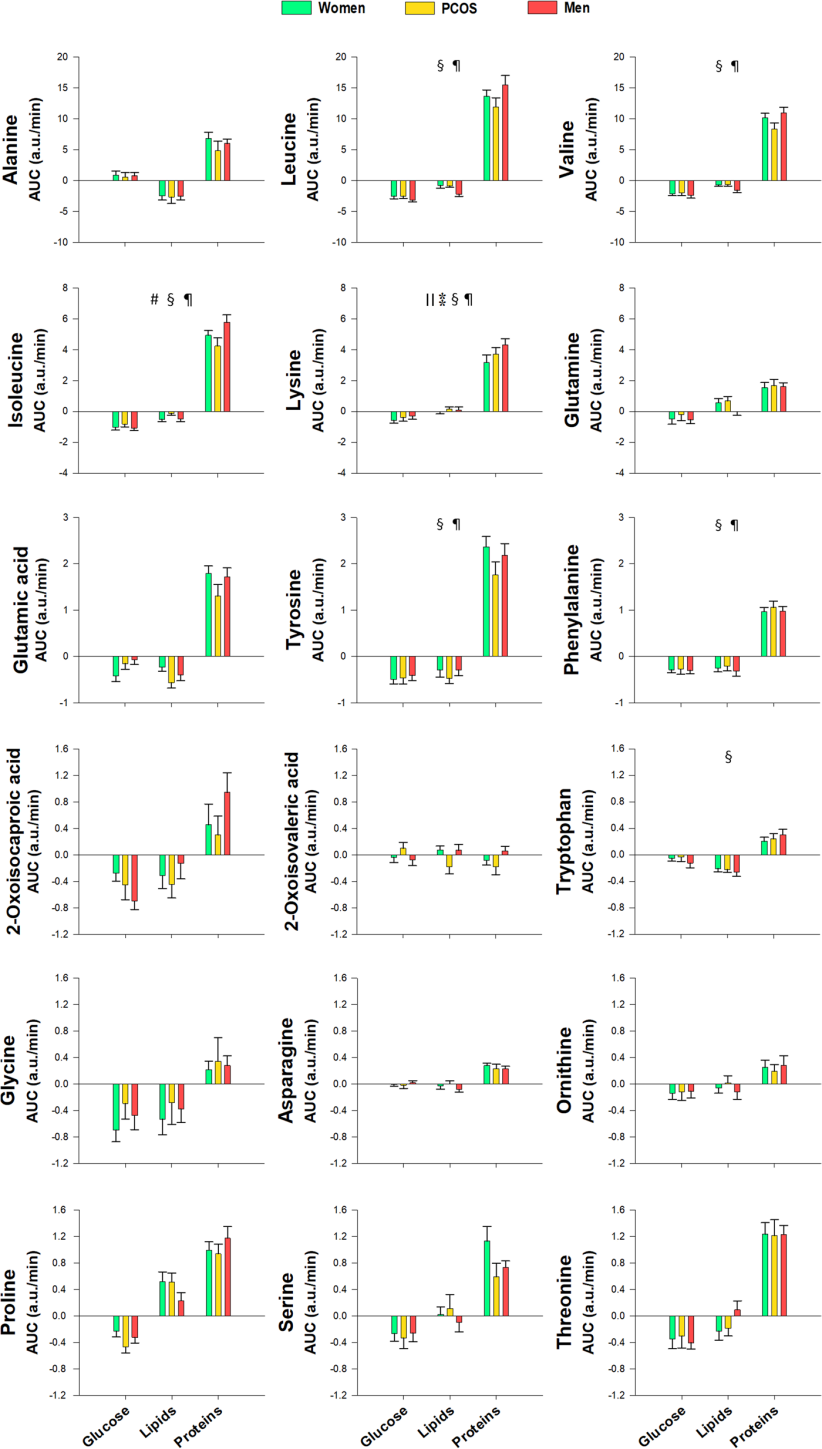

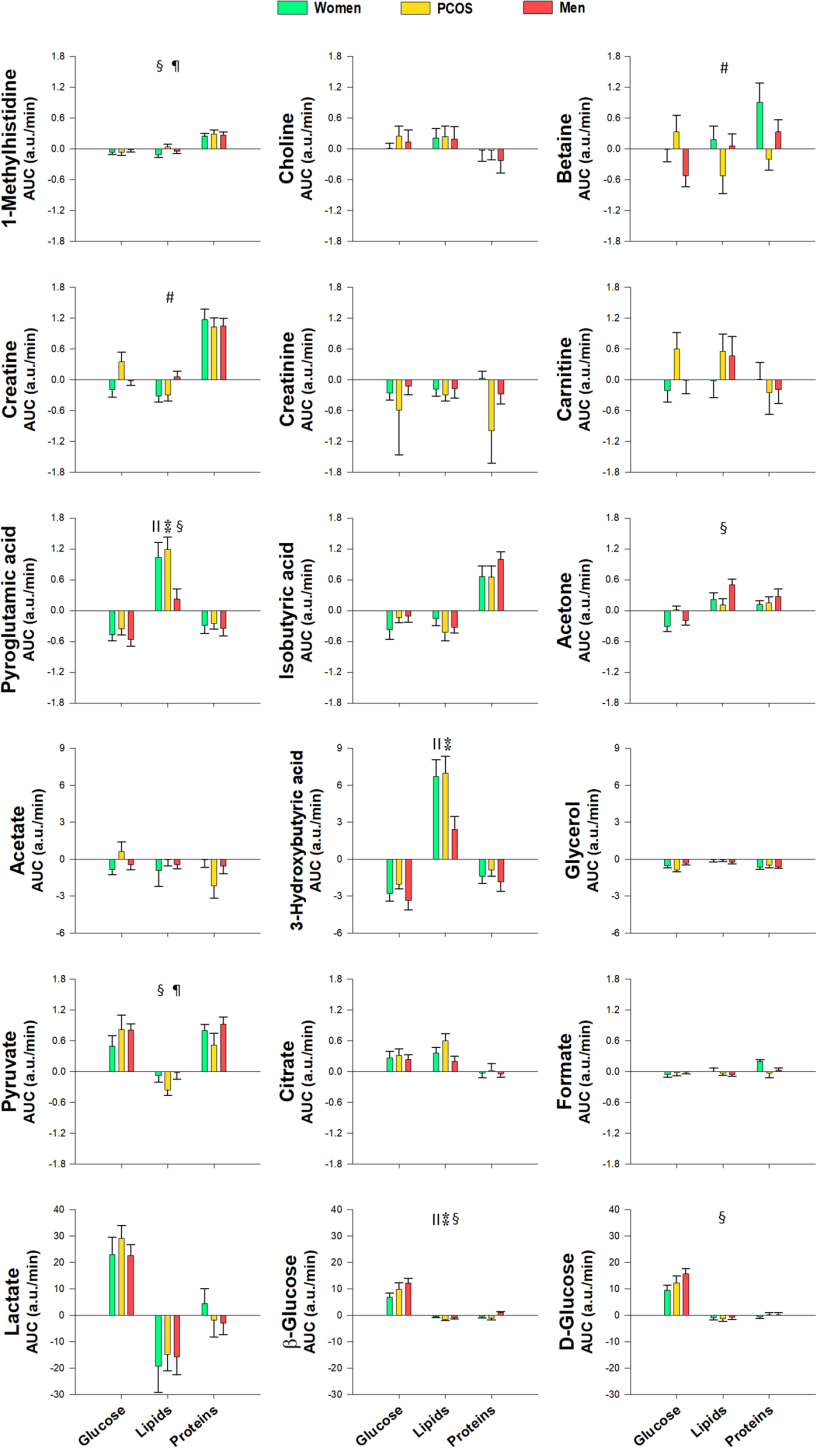
Proton nuclear magnetic resonance metabolomics profiling of amino acids, including aromatic and branched-chain amino acids and byproducts, and amino acid-derived metabolites, short-chain fatty acids, ketone bodies and carbohydrates, in response to macronutrient challenges as a function of group of subjects. Data represent the AUCs (arbitrary units) as means ± SEM. Colors green, amber and red correspond to control women, women with PCOS and men, respectively. ^║^*p* < 0.05 for the differences among groups of subjects, regardless of the macronutrient ingested and obesity (^⁑^indicates an effect of sex consisting of differences between men and control women); ^#^*p* < 0.05 for the interaction between group and macronutrient load, irrespective of obesity. ^§^*p* < 0.05 for the interaction between obesity and group of subjects, regardless of the macronutrient ingested. ^¶^*p* < 0.05 for the triple interaction among obesity, group and macronutrient load. These interactions are described in the main text and in Table 2

Regardless of the macronutrient being ingested, the only statistically significant differences found between groups consisted of increased lysine and β-glucose and decreased pyroglutamic and 3-hydroxybutyric acids in control men compared with control women (Table 2 and Fig. 4). No differences were found between women with PCOS and control women when considering postprandial responses irrespective of the macronutrient challenge.

### Interactions between independent variables

We found several interactions among the type of macronutrient administered and the effects of obesity and/or group of subjects even in metabolites on which these independent variables exerted no statistically significant effects (Table 2 and Figs. 3 and 4).

During the oral protein challenge, branched-chain amino acids (leucine, isoleucine and valine) showed a blunted postprandial increase in obese men compared with non-obese males, who also had larger increases in these amino acids compared with non-obese women. In addition, after protein intake, lysine and 1-methylhistidine showed smaller increases in obese men compared with obese women regardless of PCOS, whereas tyrosine showed a graded effect of sex and PCOS in obese individuals, consisting of a larger increase in obese control women compared with non-obese ones, no differences in women with PCOS irrespective of obesity status, and smaller increases in obese men compared with their non-obese counterparts (Table 2). Overall, this may be consistent with tissue insulin resistance being aggravated by obesity, and that such a mechanism was more important in men than in women possibly because of their larger muscle mass, with women with PCOS showing intermediate changes in some metabolites.

After the oral glucose challenge, obesity blunted the decreases observed in lysine and phenylalanine, an effect that was restricted to women in some cases. In addition, after glucose intake, obesity amplified the increases in d-glucose, β-glucose and pyruvate, but this effect was limited to obese women with PCOS compared with their non-obese counterparts, in whom the increase of these metabolites was blunted. On the other hand, in non-obese subjects the increase in β-glucose was larger in men than in women (Table 2).

Regardless of the macronutrient challenge (considering all macronutrient loads as a whole), we found differences between male and female controls in the postprandial response of pyroglutamic acid and acetone that were restricted to non-obese individuals (Table 2). In contrast, only obese women with PCOS showed increased leucine, lysine, phenylalanine and tryptophan compared with non-obese PCOS patients (Table 2). For betaine and creatine, women with PCOS also manifested a different response compared with control women and men depending on the type of macronutrient administered (Fig. 4 and Table 2).

## Discussion

Our present results provide new insights on the postprandial dysmetabolism associated with obesity and PCOS in young and otherwise healthy adults. To our best knowledge, this is the first time that ^1^H-NMRS profiling is applied to the study of separate oral glucose, lipid and protein challenges in obese and non-obese healthy women, women with PCOS and healthy men within a single metabolomics study. Exploring the postprandial response to macronutrient challenges allowed the identification of additional metabolic changes that might have been not observed in the fasting condition. The changes observed in the postprandial ^1^H-NMRS metabolomics profiles reported here were consistent with the leading effects of insulin during the switch from the fasting to the fed states in young adults, their sexual dimorphism, and their relative impairment in conditions characterized by insulin resistance, such as obesity and PCOS. These sex and sex hormone-related differences should be taken into account when designing strategies to manage metabolic dysfunction in clinical practice. Unfortunately, nowadays, such strategies are applied similarly to men and women, and because screening for PCOS is not recommended by current clinical guidelines, the possible influence of PCOS is rarely taken into account when managing women with obesity or type 2 diabetes.

The main effects of insulin during the postprandial phase include inhibition of proteolysis, inhibition of lipolysis, inhibition of ketogenesis, stimulation of glycolysis in muscle and adipose tissue, and stimulation of glycogen synthesis [40], with oral glucose being the major stimulus for insulin secretion, followed at a long distance by proteins, and with lipid ingestion showing much smaller effects on insulin release [41].

These effects may explain most of the changes in the ^1^H-NMRS metabolome observed after the oral glucose challenge, whereas the absorption of amino acids would explain the large increases observed in most amino acids and derivatives after protein ingestion. Lipid ingestion did not stimulate insulin secretion in our young adults and, other than maintaining glycerol levels and increasing several ketone bodies, shared with glucose intake the decrease in many amino acids and derivatives, and with proteins the absence of relevant changes in carbohydrates.

Metabolic flexibility is the ability to respond or adapt to conditional changes in metabolic demand, particularly regarding fuel selection at the tissue level during the transition from fasting to fed states [42]. Skeletal muscle and adipose tissue play a major role in metabolic flexibility in humans, which is impaired in conditions characterized by insulin resistance [42]. Our results indicated several effects of obesity, male sex and PCOS on ^1^H-NMRS metabolomics profiles suggesting that some degree of metabolic inflexibility is associated with these conditions. The higher postprandial d-glucose, β-glucose and pyruvate levels observed in obese subjects compared with their non-obese counterparts—regardless of the macronutrient being administered—may indicate less effective stimulation of muscle and adipose tissue glycolysis in the former as a result of their greater insulin resistance.

Our results are consistent with previous studies that showed higher pyruvate levels in obese individuals compared with lean adolescents during an oral glucose tolerance test [43]. These levels could be explained, at least in part, by an insufficient metabolization of pyruvate for ATP conversion through the TCA cycle as a consequence of decreased pyruvate dehydrogenase activity in obesity [44]. In addition, insulin resistance impairs the pyruvate dehydrogenase complex in muscle [45]. Increased postprandial pyruvate levels may also account from the conversion of lactate to pyruvate by the enzyme lactate dehydrogenase in the liver [46]. In addition, pyruvate would be converted to glucose by gluconeogenesis, given that in insulin resistant states hepatic gluconeogenesis is not inhibited by insulin [40, 41]. Our present finding of increased postprandial β-glucose and d-glucose in obese individuals, and mainly in PCOS women, support this explanation.

Sexual dimorphism [9, 11] and specific PCOS effects [20, 23] influence postprandial dysmetabolism [16]. Hence, the differences reported here in the ^1^H-NMRS metabolomics profiles that depended on sex, PCOS and their interactions with obesity merit a detailed description. In addition, although interactions between three independent variables must be always interpreted with caution, particularly when comparing relatively small groups, such as ours, present results suggest that the impact of obesity on postprandial metabolome is worse in men compared with women, with patients with PCOS sharing some characteristics with men.

The lower increase in 3-hydroxybutyric acid found in men compared with women mainly after lipid ingestion may suggest reduced lipid oxidation in the former, as the acetyl-CoA derived from beta-oxidation of fatty acids are one of the main sources for ketogenesis. In agreement, enhanced postprandial hepatic free-fatty acid oxidation and larger ketogenic capacity characterize female mammals [47, 48]. This could be related to an excess of branched-chain amino acids as substrate actually blocking the oxidation capacity of mitochondria, resulting also in an impairment of the oxidation of lipid substrates in muscle [49]. In addition, mounting evidence shows fundamental sex differences in substrate utilization as a source of fuel in mammals. Men derive their fuel energy needs preferentially from amino acids and carbohydrates, whereas women increase the mobilization of free fatty acids from fat to this avail [50, 51].

Our present results, showing that the pyruvate increase after the oral glucose challenge was exaggerated in obese women with PCOS, but was blunted in non-obese PCOS women may agree with our previous metabolomics findings of conserved insulin sensitivity in the muscle of these non-obese PCOS women, in contrast to the muscle insulin resistance of their obese counterparts [7]. Moreover, even though obesity did not influence postprandial amino acid levels when considering all subjects as a whole, when the analysis was restricted to women with PCOS we found that obese patients showed noticeable larger increases of several amino acids—leucine, lysine, phenylalanine and tryptophan—than those found in non-obese patients; such an increase would suggest a larger degree of muscle insulin resistance in obese women with PCOS compared with obese female and male controls. Insulin stimulates amino-acid uptake by the muscle to facilitate the biosynthesis of proteins [40]. In insulin resistant states, these processes would be impaired leading to increases in circulating amino acids by two mechanism, namely, decreased muscle uptake, and increased mobilization to the liver to be used as substrate for gluconeogenesis [52]. Thus, our data might support greater metabolic inflexibility in obese women with PCOS in possible association with hyperandrogenemia and muscle insulin resistance in agreement with recent reports [53–55].

The interactions observed in the increase of several branched and aromatic amino acids after protein ingestion, and the decrease of lysine and phenylalanine and increase of d-glucose, β-glucose and pyruvate after the oral glucose challenge, may be consistent with tissue insulin resistance being aggravated by obesity, and that such a mechanism was more important in men than in women, with women with PCOS showing intermediate changes in some metabolites. We observed a clear interaction between sex and obesity consisting of a common pattern of postprandial changes in several amino acids, including branched-chain and aromatic ones. In non-obese individuals, the increase in amino acid after protein intake was greater in men than in control women and patients with PCOS. However, this increase was blunted in obese men but not in obese women; the latter even presented a larger increase in some amino acids compared with their non-obese counterparts.

The profile observed in non-obese subjects may be explained by greater insulin sensitivity in muscle of women compared with men. Larger amounts of visceral and hepatic adipose tissue, in conjunction with the absence of a possible protective effect of estrogens, may contribute to higher insulin resistance in men compared with women [56–58]. In addition, increased levels of branched-chain and aromatic amino acids reduce transport of amino acids into cells [59]. Furthermore, because pyroglutamic acid is essential for the intracellular transport of free amino acids, its reduced levels may possibly contribute to higher postprandial glucose levels [60, 61]. In accordance, our data show that, within non-obese individuals, men presented with decreased postprandial pyroglutamic acid and higher β-glucose compared with female controls and women with PCOS. Moreover, consistent with its role in free amino acid intracellular transport, the general increase in amino acids after protein intake was greater in non-obese men than in non-obese control women and women with PCOS.

In contrast, the increase of numerous amino acids after protein ingestion was blunted in obese men but not in women, in which obesity even enhanced the increase of lysine and tyrosine, concordant with obesity-related insulin resistance. It is possible that the larger lean and muscle mass content and lower fat mass of men compared with women may account for this discrepancy, given that androgens increase lean and muscle mass, which may increase the demand of amino acids for protein biosynthesis [8].

Even though we did not observe differences in surrogate indexes of insulin resistance among control men, patients with PCOS and control women in this series of young adults, we may hypothesize that these small differences in the ^1^H-NMRS metabolomics profiles could reflect subtle derangements of insulin action at the tissue level. To this regard, our earlier gas chromatography–mass spectrometry metabolomics study indicated that hepatic insulin resistance was associated with PCOS even in the absence of weight excess, whereas peripheral insulin resistance developed only in the presence of obesity in these patients [7]. In addition, the finding of subtle abnormalities in ^1^H-NMRS profiles in control men compared with control women, amplified by obesity, might support the existence of sexual dimorphism in intermediate metabolism [11], with androgens possibly playing a role as suggested by the abnormalities shared among men and women with PCOS in this and other studies from our group [12–15, 23, 62]. In addition, differences in the genotype and genetic regulators, such as non-coding RNAs, including the long non-coding RNA X-inactive specific transcript, might also contribute both to sexual dimorphism and to sex-biased disorders [63, 64], such as PCOS [13, 65–67].

However, our study is not free from several limitations. We assessed targeted metabolomics assaying only a subset of metabolites in serum, thus precluding a broader view of the postprandial metabolomics picture and its proper interpretation, since blood metabolome may not necessarily reflect tissue-specific abnormalities. In addition, we used a semi-quantitative ^1^H-NMRS analysis that may need validation by fully quantitative metabolomics assays. The sample size of our subgroups was relatively small and was calculated with another objective in mind, thus precluding detection of smaller effects of obesity, sex and PCOS on the postprandial metabolome. Moreover, unlike earlier studies, our results derive from a population of young healthy adults in whom overt metabolic dysfunction is rare, and the women with PCOS in our series showed the classic hyperandrogenic phenotype, precluding extrapolation of the results to milder non-hyperandrogenic phenotypes of the syndrome. Women with PCOS differed from controls mostly in their hyperandrogenic background but not in abnormalities of carbohydrate metabolism or lipid profiles. In addition, a modest caloric intake of 300 kcal might have not been enough to induce more noticeable changes and our lipid load was mainly composed by non-saturated fats. Besides, the order of macronutrient challenges was not randomized, yet the possibility of carry-over effects was minimized by conducting the oral loads in alternate days. Finally, we could not standardize diet for more than 3 days before sampling and did not use food diaries to address long-term differences in diet among the subjects; these as factors may impact gut microbiota, which is a major contributor to the metabolome. Despite these shortcomings, the study was compensated by the homogeneous population studied in terms of age and BMI, a recommendation to follow the same diet before protocol starting, the quality of all procedures used in the challenges, the administration of exactly the same number of calories in the three oral loads, and the use of state-of-the-art ^1^H-NMRS metabolomics techniques.

## Perspectives and significance/conclusions

In summary, serum metabolomics profiling by ^1^H-NMRS, driven by the central role of postprandial insulin secretion and its effects, suggested sexual dimorphism in the responses to glucose, lipid and protein challenges, with obesity and, to a lesser extent PCOS, exerting modifying roles. Hence, obesity impaired metabolic flexibility in young adults, yet sex and sex hormones also influenced the regulation of postprandial metabolism.

## Data Availability

The data sets used and/or analyzed during the current study are available from the corresponding author on reasonable request.

## Abbreviations

Δ^4^A: Androstenedione
^1^H-NMRS: Proton nuclear magnetic resonance spectrometry
AUC: Area under the curve
BMI: Body mass index
DHEAS: Dehydroepiandrosterone-sulfate
*E*_2_: Total estradiol
GLM: General linear model
HDL: High-density lipoprotein
HOMA-IR: Homeostasis model assessment of insulin resistance
hsCRP: High-sensitivity C-reactive protein
ISI: Composite insulin sensitivity index
kcal: Kilocalories
OGTT: Oral glucose tolerance test
PCOS: Polycystic ovary syndrome
SHBG: Sex hormone binding globulin
*T*: Total testosterone
TCA: Tricarboxylic acid
WC: Waist circumference
WHR: Waist to hip ratio
